# Corrigendum: miR-218 is essential to establish motor neuron fate as a downstream effector of Isl1–Lhx3

**DOI:** 10.1038/ncomms9227

**Published:** 2015-08-21

**Authors:** Karen P. Thiebes, Heejin Nam, Xiaolu A. Cambronne, Rongkun Shen, Stacey M. Glasgow, Hyong-Ho Cho, Ji-sun Kwon, Richard H. Goodman, Jae W. Lee, Seunghee Lee, Soo-Kyung Lee

Nature Communications 6: Article number: 7718 (2015); 10.1038/ncomms8718 Published: 07272014; Updated: 08212015.

The financial support for this Article was not fully acknowledged. The second sentence of the Acknowledgements should have read: This research was supported by grants from NIH/NINDS (R01 NS054941), the March of Dimes Foundation and the Christopher and Dana Reeve Foundation (to S.-K.L.), NIH/NIDDK (R01 DK064678) (to J.W.L.), NIH/NIDDK (R01 DK103661, to S.-K.L. and J.W.L.), NIH/NIMH (R01 MH094416, to R.H.G.) and National Research Foundation of Korea (NRF) grants for Basic Science Research Program (2012R1A1A1001749), the Bio & Medical Technology Development Program (2012M3A9C6050508) funded by the Korean government (MEST) and for the Global Core Research Center (GCRC) funded by the Korean government (MSIP)(2011-0030001) and Aspiring Researcher Program of Seoul National University in 2014 (to S.L.).

